# Measurement Performance of Activity Measurements with Newer Generation of Apple Watch in Wheelchair Users with Spinal Cord Injury

**DOI:** 10.1055/s-0041-1740236

**Published:** 2021-12-02

**Authors:** Nils-Hendrik Benning, Petra Knaup, Rüdiger Rupp

**Affiliations:** 1 Institute of Medical Informatics, Heidelberg University Hospital, Heidelberg, Germany; 2 Spinal Cord Injury Center, Heidelberg University Hospital, Heidelberg, Germany

**Keywords:** spinal cord injuries, exercise, fitness trackers, activity tracking

## Abstract

**Background** The level of physical activity (PA) of people with spinal cord injury (SCI) has an impact on long-term complications. Currently, PA is mostly assessed by interviews. Wearable activity trackers are promising tools to objectively measure PA under everyday conditions. The only off-the-shelf, wearable activity tracker with specific measures for wheelchair users is the Apple Watch.

**Objectives** This study analyzes the measurement performance of Apple Watch Series 4 for wheelchair users and compares it with an earlier generation of the device.

**Methods** Fifteen participants with subacute SCI during their first in-patient phase followed a test course using their wheelchair. The number of wheelchair pushes was counted manually by visual inspection and with the Apple Watch. Difference between the Apple Watch and the rater was analyzed with mean absolute percent error (MAPE) and a Bland–Altman plot. To compare the measurement error of Series 4 and an older generation of the device a *t* -test was calculated using data for Series 1 from a former study.

**Results** The average of differences was 12.33 pushes ( *n* = 15), whereas participants pushed the wheelchair 138.4 times on average (range 86–271 pushes). The range of difference and the Bland–Altman plot indicate an overestimation by Apple Watch. MAPE is 9.20% and the *t* -test, testing for an effect of Series 4 on the percentage of error compared with Series 1, was significant with *p* < 0.05.

**Conclusion** Series 4 shows a significant improvement in measurement performance compared with Series 1. Series 4 can be considered as a promising data source to capture the number of wheelchair pushes on even grounds. Future research should analyze the long-term measurement performance during everyday conditions of Series 4.

## Introduction

Trauma or diseases of the central nervous system such as multiple sclerosis, stroke, or spinal cord injury (SCI) typically result in limitations up to the complete loss of the ability to walk. 1 In people with sufficient arm strength manual wheelchairs are a means for compensation of the loss of ambulation enabling some degree of mobility. Additionally, manual wheelchair use represents the primary type of physical activity (PA) in people with paralysis of the lower extremities. Wheelchair users show different amounts of PA, depending on several personal factors such as body function, employment status, and psychological factors. 2 The lack of PA can reduce physical fitness and increases the risks of secondary chronic complications in particular in respect to the cardiometabolic health. 3 Studies in SCI have reported that regular PA can lead to an improved health condition by reducing cardiovascular risks, 4 pain, 5 the risk for shoulder pain and depressive symptoms, 6 and the risk for developing pressure injuries. 7 On the other hand, it is known from literature that too high amounts of PA in wheelchair athletes result in severe shoulder complications including chronic pain. 8 However, long-term studies correlating the amount of PA with the development of musculoskeletal complications are missing. A key prerequisite for this investigation is the collection of objective data on the individual amount of PA. The availability of such data would enable further studies on the influence of PA on health-related outcomes.

One approach to this is to use activity trackers for continuous measurement of PA. Activity trackers, based on inertial measurements units (i.e., accelerometer and gyroscope), are widespread tools to generate reliable measures for PA in people without disabilities. 9 However, there is less evidence for the validity of activity trackers for wheelchair use than for ambulatory use. Beside proprietary activity trackers or wheel turn measurement devices the only off-the-shelf, wearable activity tracker with specific measures (number of wheelchair pushes, travelled distance, and activity energy estimation) for wheelchair users is the Apple Watch. Apple Inc. put several Apple Watch hardware revisions on the market. 10 With Series 4, Apple Inc. has released a hardware revision with a new sensor platform, incorporating a more precise accelerometer and gyroscope. 11

Existing studies have been performed with early generation (pre-Series 4) Apple Watches. Series 1 has undergone two studies on the measurement error for wheelchair users, indicating a percentage of absolute error of 13.5% with a range of 1 to 22% (depending on activity types such as treadmill driving, arm cycle ergometry, or overground driving). 12 13 To establish ground truth, existing studies counted pushes retrospectively by observing videos in normal speed 12 or prospectively during direct observation. 13

## Objectives

The aim of this study was to (1) analyze the absolute measurement error of Apple Watch Series 4 for tracking wheelchair pushes in people with subacute SCI while self-paced driving a defined course in a SCI center's gym and to (2) evaluate its measurement error on a group level with data from another study using an older version of the Apple Watch. 13

The report on this study was created following the Guidelines for Reporting Reliability and Agreement Studies. 14

## Methods

### Materials

To establish the ground truth of push count, a digital tally counter was used by trained raters. An Apple Watch Series 4 with WatchOS 6.2.6 was used for the study. To activate and configure the Apple Watch an Apple iPhone 7 with iOS 13.5.1 was used.

### Participants

We included 15 participants with subacute SCI (< 12 months), who were at least 18 years old, via convenience sample. Thirteen participants were diagnosed with paraplegia and two participants were diagnosed with tetraplegia. Three participants were female (20%), and 12 participants were male (80%). The average age was M = 45.0 years (standard deviation = 15.8 years), ranging from 20 to 74 years. All participants wore the activity tracker on the left arm. All study participants had to be able to use a manual wheelchair, be able to follow the study procedures, and to give informed consent. The participants used their own wheelchairs which were adapted to the individual's anatomy. They were recruited at the SCI center of Heidelberg University Hospital, while being in the inpatient rehabilitation phase. The demographic and SCI-related states of the participants at the time of the examination are shown in Table 1 . All investigations were performed from July to October 2020. Written informed consent was obtained prior to study inclusion. The study has been approved by the ethics committee of Heidelberg University.

**Table 1:** Demographic and SCI-related data of study participants

ID	Sex	Age	Neurological level of injury	AIS	Arm (watch)
1	M	30–35	L2	B	L
2	F	70–75	T10	D	L
3	M	50–55	L1	D	L
4	M	30–35	T12	C	L
5	M	60–65	T12	A	L
6	M	40–45	L4	D	L
7	M	40–45	L1	A	L
8	M	20–25	C8	A	L
9	M	50–55	T12	D	L
10	F	45–49	T4	D	L
11	M	20–25	C6	B	L
12	M	55–59	T5	A	L
13	M	30–35	L1	C	L
14	F	65–69	L1	D	L
15	M	35–39	T4	A	L

Abbreviations: AIS, American Spinal Injury Association (ASIA) Impairment Scale 15 ; SCI, spinal cord injury.

### Setting

**Fig. 1:**
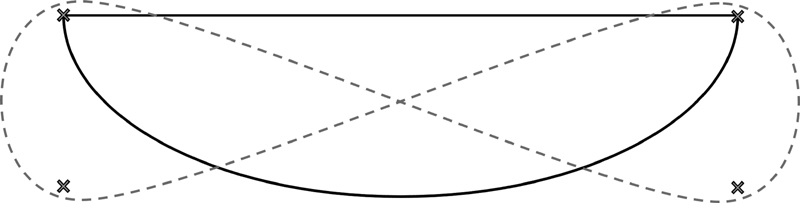
Schematic course layout of Part A (solid black, 93.6 m) and Part B (dashed gray, 94.8 m). Crosses represent pylons.

**Fig. 2:**
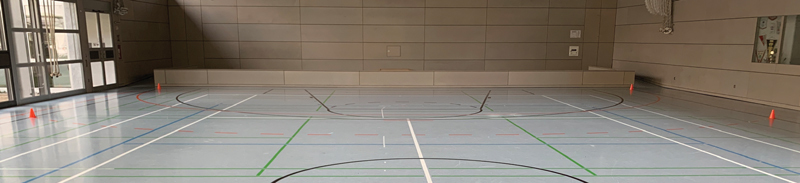
Photo of the course. The orange line marks the semicircle of Part A.

A two-part test course in a gym with a matched number of right and left turns was used. The course included curves with narrow and wide radius. This was done to include phases, during which participants had to use one hand for pushing one wheel and one hand for stopping the other. The course consisted of two parts: Part A had the shape of a semicircle ( Fig. 1 , black line) and Part B had the shape of an eight ( Fig. 1 , gray line). Part A was marked by the gym's ground lines and two pylons. Part B was marked with four pylons. The distance of Part A was 93.6 m, and the distance of Part B was 94.8 m. Fig. 2 shows a photo of the setting.

### Procedure

**Fig. 3:**
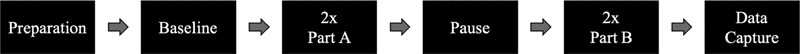
Overview of an individual experiment.

The procedure consisted of five steps, as shown in Fig. 3 . First, participants were introduced verbally to the setting and materials, including Apple Watch, were prepared. Next, baseline data from Apple Watch was captured using the “Activity” app. Then, participants were asked to drive Part A of the course twice. After a short pause, participants were asked to drive Part B of the course twice. While driving the course, a rater manually counted the pushes. In total, two raters were involved, one of which trained the other to use the same systematic definition of a push. Finally, measurement data from Apple Watch was captured, using the “Activity” app again.

#### Preparation

Participants were asked to wear the Apple Watch on their nondominant arm (i.e., left arm for right-handed participants) as this reflects the typical behavior given by the crown orientation of classical watches. In all test series the close fit of the watch was ensured by a visual and haptic check. Participants were asked verbally for their birthdate, sex, body height, and weight. These data were entered on the fly into Apple Watch app, which is supposed to increase measurement performance. 16 The data was entered at the “Health Profile” menu in the “Health” settings of Apple Watch app.

#### Baseline

Participants were asked to rest their arms on their thighs, avoiding any unnecessary movements of the Apple Watch. They had to rest their arms for at least 60 seconds, to ensure stable baseline values. After 60 seconds the initial push count of Apple Watch was documented from the “Activity” app on the watch itself. For sample description the age, sex, and nondominant arm of the participant were recorded on the fly.

#### Part A

The rater was positioned on the side of the nondominant arm of the participants and asked them to start the course at a speed comfortable. A second person guided the participants through the test course by walking in front of them, keeping a distance of at least 3 m at any time. The rater counted the wheelchair pushes of each participant using a digital tally counter.

Push counting was conducted analogously to the preliminary study. 17 During the preparation of the preliminary study, we conducted initial experiments, which indicated that Apple Watch does not count backward pushes. Thus, a push is defined by one forward movement with the hand of the arm wearing the Apple Watch. A forward movement was only considered as a push, if the participant held the handlebar of the wheel for approximately ≥ 20 degrees of the wheel's radius, while rolling forward (as seen in Benning et al 17 ). Pushes were counted at all speeds. Potential backward movements for maneuvering were not counted as pushes. In total, two raters were involved. For training of the rater, who was not involved in the preliminary study, 17 parts of the course have been counted in parallel to the rater, who was involved in the preliminary study. The new rater was considered as trained after he counted the same number of pushes in both, Part A and Part B of the course. The preliminary study has indicated that manual counting of wheelchair pushes is an appropriate method (intraclass correlation coefficient [ICC] = 0.981, 95% confidence interval: 0.96 < ICC < 0.99). 17 Participants were guided through Part A of the course twice, so they covered it clockwise and counterclockwise.

#### Pause

If participants performed the course correctly, they were asked to stop at the pylon for at least 5 seconds. Arm movement was prohibited by holding the handlebars still.

#### Part B

Participants were asked to complete Part B of the test course. While driving, the rater continued counting the pushes as mentioned above and participants were guided through the course by the examiner twice. After completing the first iteration of Part B, participants were asked to change the direction by driving around one of the pylons.

#### Data Capture

After completing the course participants were asked to rest their arms for 60 seconds on the thighs. The push counts reported by the rater were documented on the fly during the experiment. After the 60 seconds, the push count of the Apple Watch was recorded a second time. The delay is to ensure that the background calculation of pushes is completed and displayed in the “Activity” app on Apple Watch.

### Analysis

We considered the manual count of the wheelchair pushes of the rater as ground truth. The number of pushes captured by the Apple Watch for both phases was calculated by subtracting the push count at the end of baseline from the push count after completing the whole course. We calculated for each participant the difference to ground truth and calculated the grand average over all participants. Our key criterion to assess the measurement error of Apple Watch Series 4 is a representation independent from the total push count: The mean absolute percent error (MAPE). MAPE is calculated by dividing the sum of absolute differences by the sum of manually counted pushes. A Bland–Altman plot was created to examine data for systematic errors.

We also compared the measurement error of the Series 4 model with a Series 1 model. Therefore, Glasheen et al kindly provided us with the data from their study 13 which holds comparable data sets of wheelchair users in the category “Obstacle course – figure 8” ( *n* = 13). 13 To test the Apple Watch Series 4 improvements for a significant effect, a Welch two-sample *t* -test was used. The null hypothesis is the absence of an effect on the error rate. The alternative hypothesis is an effect on the error rate. To assess the statistical power of the model, a post hoc power analysis was conducted with the help of G*Power 3.1.

### Ethical Considerations

The ethics committee of Heidelberg University (chair: Prof. Dr. med. Dr. h. c. T. Strowitzki), Heidelberg, Germany, approved the study under reference number S-084/2020. The procedures followed were in accordance with the Helsinki Declaration of 1975, as revised 2013. All participants gave written informed consent before enrolment.

## Results

### Absolute and Relative Deviation from Ground Truth

The total number of manually counted wheelchair pushes per participant ranged between 86 and 271. We analyzed the range of differences of the pushes counted by Apple Watch compared with the pushes counted manually. Further, the average of these differences and the MAPE were calculated. The average number of manually counted pushes is 138.4. The differences of the pushes counted by Apple Watch compared with the pushes counted manually range from –3 to +38 pushes for all test series. The mean difference is 12.33 pushes. The MAPE represents the overall share of the absolute differences in manually counted pushes and is 9.20%.

### Bland–Altman Plots for Systematic Examination of Deviations from Ground Truth

**Fig. 4:**
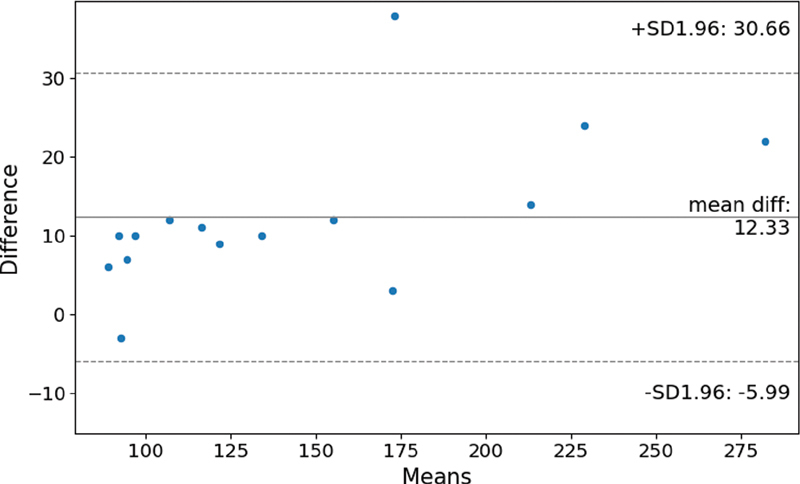
Bland–Altman plot presenting the difference between Apple Watch count and manual count against the means of Apple Watch and manual count.

The Bland–Altman plot shows differences, which were calculated by subtracting manually counted pushes from Apple Watch recorded pushes. Thus, negative differences represent underestimations of the Apple Watch and positive differences represent overestimations. The Bland–Altman plot can be found in Fig. 4 . The mean difference is 12.33 pushes, representing the average bias of push counts by Apple Watch. The 95% limits of agreement, reflecting the fluctuations around the mean difference, are 30.66 pushes and –5.99 pushes.

### Comparison of Apple Watch Series 4 and Series 1

With the data from Glasheen et al we compared the MAPE of Apple Watch Series 1 and Series 4. 13 The MAPE was compared by subtracting Series 1 MAPE (20.62%) from Series 4 MAPE (9.20%) on a group level. Thus, a positive difference represents a reduced error and a negative difference an increased error for Series 4. We found a difference of 11.42%.

The effect of the new Apple Watch generation was analyzed by comparing the percentage of errors of the test series in the study of Glasheen et al and our study. 13 Homogeneity of variances was assessed using a Levene's test with median as center. The test was significant with *F* (1,26) = 8.978, *p* = 0.006. Thus, the variances can be considered as inhomogeneous. Based on the inhomogeneity of variances we calculated a Welch two-sample *t* -test, to test for a true difference of percentage of errors between Series 1 and Series 4. The *t* -test was significant with *t* (16.143) = 3.011, *p* = 0.008. Thus, Apple Watch Series 4 has a significant effect on percentage of error for push-counting compared with Series 1. The post hoc power analysis was performed for an effect size of *d* = 1.246, *α* = 0.05 and the sample sizes of *n*_Series1_ = 13 and *n*_Series4_ = 15. The power of the model is 1– *β* = 0.940.

## Discussion

### Discussion of Results

The differences from ground truth for Apple Watch Series 4 result in a MAPE of 9.2%. The range of differences has a maximum value more than 10 times as high as the minimum value and the Bland–Altman plot shows, with one exception, only overestimates. Thus, Apple Watch seems to have a general tendency to overestimate the push count.

This effect compares well to the study from Glasheen et al, who also found a systematic overestimation of Series 1 with MAPE values ranging between 1 and 22%, depending on the type of propulsion (wheelchair treadmill, arm cycle ergometer, gym courses). 13

However, Karinharju et al found a general underestimation for Series 1 with a MAPE of 13.5%. 12 This difference could be explained by a different definition of manually counted pushes: Karinharju et al defined the push count by the number of times where the wheelchair user moves the wheelchair by applying force to the handlebar of the wheel. 12 Thus, this definition includes backward movements for maneuvering, which leads to higher manual counts. Furthermore, Karinharju et al do not state if pushes were counted just if the arm where the Apple Watch was worn was used or even if just the arm where the Apple Watch was not worn was used to propel the wheelchair. This would further increase manual counts. In our study, a wheelchair push was defined by forward movements of the hand while holding the wheelchair's handlebar. We only counted pushes from the arm wearing Apple Watch, because it seems technically challenging to track pushes from the arm not wearing an activity tracker. We interpret the unilateral measurements as a technical limitation of wrist-worn activity trackers which should be considered in study protocols regarding the manual observation. In typical everyday use we do not consider this as a relevant limitation, as physical movement is achieved using both arms equally by wheelchair users.

The Bland–Altman plot shows a tendency for higher overestimations for higher push counts on the course. From our experience a higher push count indicates a higher movement impairment and thus a less efficient use of the wheelchair. Following the Bland–Altman plot, Apple Watch is subjected to have a higher risk for measurement errors in this group of wheelchair users.

Our *t* -test result indicates a significant effect on measurement error by the new Apple Watch generation Series 4 compared with Apple Watch Series 1. Considering the MAPE for both generations in comparable test scenarios (20.62% for Series 1 13 and 9.20% for Series 4), the effect of Series 4 can be considered as an improvement of measurement performance. The MAPE observed in this study is also lower than our observations in a preliminary study with nondisabled people using a wheelchair: In the preliminary study we found a MAPE of 13.90% for an individually calibrated Apple Watch Series 4 with unexperienced wheelchair users. 17 This can be explained by altered movement patterns in unexperienced wheelchair users compared with more experienced wheelchair users, which are the intended users of the Apple Watch wheelchair mode.

Existing studies on PA in people with SCI used interview formats for PA data capture. 3 4 5 6 7 8 There are well-established methods like the interview-based 3-day recall format Physical Activity Recall Assessment for People with Spinal Cord injury (PARA-SCI) 18 or the 7-day recall format Leisure Time Physical Activity Questionnaire for People with Spinal Cord Injury (LTPAQ-SCI). 19 However, depending on the necessary precision of PA data, interview formats bind human resources to conduct the interviews: LTPAQ-SCI causes a pure interview time of less than 5 minutes for each participant in a study, 19 PARA-SCI 60 to 90 minutes. 20 Apart from the time necessary, interview formats always depend on the answers of participants. This can cause a limited adherence and higher dropout rates because participants might not accept the time effort necessary to participate in a study. Despite the systematic nature of questions from PARA-SCI, interview formats have a risk for subjective estimates from participants. The potential for such a biased self-estimate can be seen in a study by Ma et al, who compared PA captured by PARA-SCI with PA captured by sensors and found substantial differences. 21 Apart from this, interview formats are limited in terms of the examined period of time: Whereas the PARA-SCI is able to cover a period of 3 days 18 and LTPAQ-SCI 1 week, 19 typical effects correlated with PA occur years after the inpatient rehabilitation phase. 22 Nevertheless, patient-reported measures are complementary to device-based measures. Their combined analysis offers the possibility for gaining new insights into correlations and interdependencies. With regard to the examined feature type of the number of wheelchair pushes this applies to the perceived level of effort and exertion (e.g., measured by the Borg scale) for their execution, for example.

The study results show that a reliable measurement of the number of wheelchair pushes is feasible in an inpatient, hospital setting, eliminating limitations of interview formats. Use cases for this can be found while people are in-patients during their first phase of rehabilitation. Apple Watch may also support people in the phase after discharge, where assessment of changes of PA and orientation on normal amounts on PA can be helpful. Wheelchair pushes can be considered as one form of PA in people with SCI; however, there are also other substantial forms of PA without active wheelchair movement, for example, everyday tasks such as housekeeping and training such as cranking or dumbbell exercise. Despite their limitations, abovementioned interview formats such as PARA-SCI, 18 actually capture all kinds of PA. Other alternative approaches are wheelchair-mounted systems, such as the SmartWheel 23 or smartphone apps, using the smartphone built-in accelerometers 24 to estimate PA of persons with SCI. Especially the SmartWheel promises a higher measurement performance regarding the travelled distance and push forces as measure of PA. However, the SmartWheel is a quite expensive device not compatible with every wheelchair model. The smartphone-internal sensor-based solutions have difficulties to differentiate between active and passive propulsion.

### Limitations

The study sample is not representative with respect to the sex and dominant arms of participants for the wheelchair user population. 25 Compared with other studies, the sample size is reasonable for an evaluation of the measurement error of Apple Watch Series 4; however, an increased sample size would be desirable to validate the measurement error of Apple Watch Series 4 on a larger scale. The course in our experiments does not represent a real-world scenario but rather an artificial laboratory setting. However, this setting enabled us to recruit patients with subacute SCI, who would not be able to complete a real-world outdoor course in many cases. Activity tracking is especially important for these people, as they have to learn about their personal amounts of PA in the context of SCI.

While wheelchair pushes are not an accurate representation of PA, they can be used analogously to step counts for ambulatory persons. 26 They provide a measurable indicator, which is quite easy to interpret by people with SCI and thus may motivate them to lead a healthier lifestyle.

Our comparison with the results from Glasheen et al was limited to their protocol phase “Obstacle course – figure 8” as this is the most comparable part of their study protocol to our course. This comparability is especially valid for Part B of our course: The form compares well (shape of an eight), as well as the obstacles (pylons) and the length (94.8 m and approximate 100 m). 13 However, including Part A in this comparison limits comparability of the studies. The presented study has more restrictive inclusion criteria (especially it is limited to people with SCI), whereas Glasheen et al included eight people with SCI but also included seven people with other diseases, namely cerebral palsy, hemorrhagic stroke, traumatic brain injury, degenerative disc disease, and spina bifida. 13

Based on experiences from existing studies on the measurement performance of activity trackers for people who ambulate, measurement performance might be lower for self-paced movements. 27 Although it is unclear to what extent the results on the measurement performance of pedometers can be transferred to wheelchair push counters, it would be of interest to investigate measurement performance with different defined speeds.

In our study we used Apple Watch in uncalibrated mode. The device allows for an individual calibration to the movement patterns of the wheelchair user, which is subjected to increase measurement performance. 28 Even though our preliminary study has shown that the effect is neglectable within equivalence bounds of ± 15%, further research should examine the effect of the calibration for wheelchair users with SCI. 17 However, calibration extends the procedure by more than 20 minutes and requires GPS coverage, making it necessary to perform examinations in an outdoor setting. We expect this to have a negative impact on participant recruitment.

For our materials we used Apple Watch Series 4, which is not the latest model from Apple at the time of publication. Newer device and software generations could have a positive effect on measurement performance. Anyhow, Apple did not mention further upgrades of the sensor platform in post-Series 4 generations in public communications.

In general, we observed an increased measurement performance for the Apple Watch Series 4. Despite general limitations of the Apple Watch such as the measurement limited to one arm and exclusive push counting while rolling forward, we consider newer Apple Watch generations as a promising data source for data-driven research on PA under everyday conditions in persons with SCI. While push counting with the abovementioned limitations does not fully represent the PA of a person, it provides people with SCI with an easily interpretable measure for their PA. If push counts, captured by Apple Watch, are to be compared with push counts from other activity trackers, it should be ensured that the pushes from other activity trackers are captured in the same way as Apple Watch does.

### Future Outlook

As the Bland–Altman plots have shown a tendency for higher overestimations for higher push counts in the course, the measurement performance should be analyzed for PA-level stratified subgroups. From our experience, a higher push count on the course is connected with more severe motor impairment. Thus, future studies should compare the measurement performance in groups defined by lesion level (people with paraplegia, vs. tetraplegia).

The measurement performance might offer the potential to use the Apple Watch for SCI research. Its data would enable a more detailed analysis of PA-based determinants on the development or prevention of complications and ultimately on the quality of life of people with SCI. To examine long-term effects of PA on health outcomes, an integration of the data into a life-long patient-centered registry (ParaReg) is a promising approach. 29 In a first analysis the baseline at discharge should be used as a reference to investigate the individual course of PA in the form of wheelchair pushes over time. Since we do not know yet how accurately wheelchair pushes measure PA, this should be a question of further research. Another important research question is, how wheelchair pushes correlate with long-term SCI-related complications. Ultimately, evidence on the connection of PA and SCI-related complications can lead to push count recommendations for people with SCI and even PA-based prediction models for SCI-related complications, enabling precisely targeted prevention measures.

## Conclusion

Apple Watch Series 4 shows a significant improvement in measurement performance compared with Apple Watch Series 1. We observed a general overestimation for Apple Watch Series 4 with a 9.20% mean absolute percentage of error. We consider the integration of PA data, captured by Apple Watch Series 4, into medical registries as promising approach to enable researchers to analyze the impact of PA on the risk for the development of long-term complications and to ultimately refine current recommendations on the amount of PA for people with SCI on an individual level.
